# Inflammatory Reaction Induced by Two Metalloproteinases Isolated from *Bothrops atrox* Venom and by Fragments Generated from the Hydrolysis of Basement Membrane Components

**DOI:** 10.3390/toxins12020096

**Published:** 2020-02-02

**Authors:** Michelle Teixeira de Almeida, Luciana Aparecida Freitas-de-Sousa, Monica Colombini, Sarah N. C. Gimenes, Eduardo S. Kitano, Eliana L. Faquim-Mauro, Solange M. T. Serrano, Ana Maria Moura-da-Silva

**Affiliations:** 1 Programa de Pós-Graduação em Ciências-Toxinologia, Instituto Butantan, São Paulo 05503-900, Brazil; michelle.almeida@butantan.gov.br; 2 Laboratório de Imunopatologia, Instituto Butantan, São Paulo 05503-900, Brazil; luciana.sousa@butantan.gov.br (L.A.F.-d.-S.); monica.colombini@butantan.gov.br (M.C.); sarah.gimenes@butantan.gov.br (S.N.C.G.); eliana.faquim@butantan.gov.br (E.L.F.-M.); 3 Laboratório de Toxinologia Aplicada, Center of Toxins, Immune-Response and Cell Signalig, CeTICS, Instituto Butantan, São Paulo 05503-900, Brazil; eduardosh.kitano@gmail.com (E.S.K.); solange.serrano@butantan.gov.br (S.M.T.S.)

**Keywords:** *Bothrops atrox*, SVMPs, metalloproteinases, basal membrane, hydrolysis, peptides, inflammation

## Abstract

Snake venom metalloproteinases (SVMPs) play an important role in local tissue damage of snakebite patients, mostly by hydrolysis of basement membrane (BM) components. We evaluated the proinflammatory activity of SVMPs Atroxlysin-Ia (ATXL) and Batroxrhagin (BATXH) from *Bothrops atrox* venom and their hydrolysis products of Matrigel. BALB/c mice were injected with SVMPs (2 μg), for assessment of paw edema and peritoneal leukocyte accumulation. Both SVMPs induced edema, representing an increase of ~70% of the paw size. Leukocyte infiltrates reached levels of 6 × 10^6^ with ATXL and 5 × 10^6^ with BATXH. TNF-α was identified in the supernatant of BATXH—or venom-stimulated MPAC cells. Incubation of Matrigel with the SVMPs generated fragments, including peptides from Laminin, identified by LC–MS/MS. The Matrigel hydrolysis peptides caused edema that increased 30% the paw size and promoted leukocyte accumulation (4–5 × 10^6^) to the peritoneal cavity, significantly higher than Matrigel control peptides 1 and 4 h after injection. Our findings suggest that ATXL and BATXH are involved in the inflammatory reaction observed in *B. atrox* envenomings by direct action on inflammatory cells or by releasing proinflammatory peptides from BM proteins that may amplify the direct action of SVMPs through activation of endogenous signaling pathways.

## 1. Introduction

Snakebites represent a major public health problem and are considered a neglected tropical disease. According to some estimates, there are 1.8 to 2.7 million ophidian envenomings in the world annually, of which about 81,000 to 138,000 result in death [1]. In Brazil, the genus *Bothrops* is responsible for the greatest number of these accidents, which are characterized by several systemic or local effects that can evolve into significant temporary or permanent disabilities. These effects are caused by a wide range of toxins present in the venoms of *Bothrops* snakes, such as serine proteinases, phospholipases A_2_ and snake venom metalloproteinases (SVMPs), which participate in different events, including inflammation [2].

Studies with venoms from *Bothrops* snakes have demonstrated their proinflammatory activity, since these venoms are capable of causing increased vascular permeability, formation of edema, recruitment of leukocytes and expression of adhesion molecules, cytokines and chemokines [3]; in such events, SVMPs play important role. SVMPs are zinc-dependent enzymes, classified in three classes, based on their precursors: the PI-class is composed of the pre-, pro- and metalloproteinase domains; PII-class of pre-, pro-, metalloproteinase and disintegrin domains; and PIII-class composed of pre-, pro-, metalloproteinase, disintegrin-like and cysteine-rich domains [4]. The PI- and PIII-classes are widely expressed in viper venoms and well characterized for their proinflammatory action, which is frequently associated with their catalytic activity [5,6,7] or with the activation of inflammatory cells as macrophages that release proinflammatory mediators [8,9]. Due to their catalytic activity, SVMPs may also have action on endogenous pro-metalloproteinases and pro-cytokines, such as pro-MMPs [10] and pro-TNF-α [11], which, upon cleavage by SVMPs, are released in their active form. However, the proinflammatory activity of these enzymes is not only due to the presence of the catalytic activity, but also to their action on cell receptors through the disintegrin-like and/or cysteine-rich domains, which can induce leukocyte recruitment and cytokine synthesis [12,13].

*Bothrops atrox* snakes are reported to be the leading cause of ophidian accidents in the Amazon region. Human envenomings are characterized in most cases by consumption coagulopathy and local damages, such as edema, pain, erythema and local hemorrhage, which are not effectively neutralized by *Bothrops* antivenom [14]. In experimental models, *B. atrox* venom displays proinflammatory activity and is capable of causing an increase in vascular permeability and an important influx of leukocytes to the site of injury, characterized by the presence of polymorphonuclear and mononuclear cells, as well as the release of the eicosanoids PGE_2_ and LTB_4_, and the cytokines TNF-α and IL-6 [15]. However, the knowledge about the contribution of each toxin class to *B. atrox* venom on proinflammatory reaction is still restricted to the isolated PI-class SVMPs. A pool of low-molecular-mass proteinases was able to induce the formation of edema and leukocyte infiltrate [16]. Considering isolated toxins, Batroxase, a PI-class SVMP isolated from the venom of *Bothrops atrox*, was capable of inducing leukocyte migration, as well as the release of proinflammatory mediators, such as IL-1β, IL-6 and PGE_2_, involved in these events [17].

PIII-class SVMPs, such as Jararhagin, isolated from the venom of *B. jararaca* [18], present hemorrhagic activity and trigger different events during the envenoming [19]. They are able to trigger the proinflammatory activity, with increased expression of cytokines, such as IL-6 and TNF-α, which are shortly degraded by the catalytic activity of SVMPs after expression in in vitro assays [12].

Recently, our group isolated two hemorrhagic SVMPs from the venom of *B. atrox* that were named Atroxlysin-Ia [20] and Batroxrhagin [21]. Batroxrhagin (BATXH) is a PIII-class SVMP structurally and functionally similar to Jararhagin, isolated from *B. jararaca* venom [21]. Atroxlysin-Ia (ATXL) is an isoform of the PI-class SVMP Atroxlysin-I, isolated from *B. atrox* Peruvian snakes [22] and is structurally different than Batroxase [23]. However, unlike the previously isolated toxins, ATXL presents a dermonecrotic activity and is capable of inducing an intense hemorrhage, in levels comparable to the PIII-class SVMP. The mechanism suggested for ATXL higher hemorrhagic and dermonecrotic action than other PI-class SVMPs was its higher efficiency to cleave Basement Membrane (BM) components as collagen IV and laminin, important structural elements that guarantees stability to BM [20]. Laminin is a glycoprotein that plays important roles in cell biology, such as cell adhesion, migration and proliferation, among other biological activities [24]. Some studies have reported different biological activities of peptides obtained from laminin degradation, for example, SIKVAV and AG73, which present the ability to regulate the expression of adhesion, migration, invasion and proteolytic activity molecules in some cell lines of carcinoma [25,26]. Thus, proteolytic hydrolysis of laminin by SVMPs could also release bioactive peptides that could interfere in cell adhesion and, consequently, in tissue organization. Rucavado et al. [27] demonstrated, by proteomic analysis, the presence of fragments of Damage Associated Molecular Patterns (DAMPs) in exudates obtained after intramuscular injection of *B. asper* venom in mice. The authors associated the presence of the peptides with the hydrolysis of extracellular matrix components and cell damage, indicating the generation of DAMPs in snakebite envenomation and its participation in inflammatory processes.

Thus, in view of the importance of proinflammatory events caused by SVMPs and the action of some bioactive peptides generated by hydrolysis of ECM components, in this study, we evaluated the proinflammatory action induced by the ATXL and BATXH, as well as by the products derived from BM degradation by these two enzymes.

## 2. Results

### 2.1. Inflammatory Reaction Induced by SVMPs

**Figure 1:**
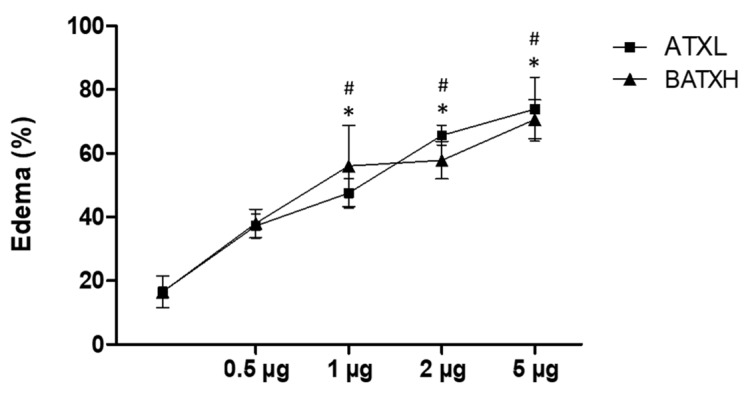
Edema induced by different doses of Atroxlysin-Ia (ATXL) and Batroxrhagin (BATXH). Mice (*n* = 6) were injected on the left paw with different protein doses (0.5, 1, 2 and 5 μg/animal) or with saline as negative control. The results are expressed as the mean ± S.E. of two independent experiments. Symbols indicate significative differences (*p* ≤ 0.05) compared to the negative control for ATXL (*) or BATXH (^#^) groups.

Our first approach was to evaluate and compare the inflammatory effects induced by the administration of ATXL and BATXH into the mouse foot paw. Edema formation was observed after injections with different doses (0.5, 1, 2 and 5 µg) of ATXL or BATXH (Figure 1). The doses of 1, 2 and 5 µg of both toxins induced edema significantly higher than the control did. The differences among doses from 1 to 5 µg, within and between each toxin group, were not statistically significant, indicating that the two-times-lower molecular mass of ATXL than BATXH would not impact the comparisons in this range of doses. The intermediate dose of 2 µg/mouse was then selected to evaluate and compare the time course of the edema induced by both toxins, since this dose was capable of inducing edema without causing hemorrhage in the animals [20].

**Figure 2:**
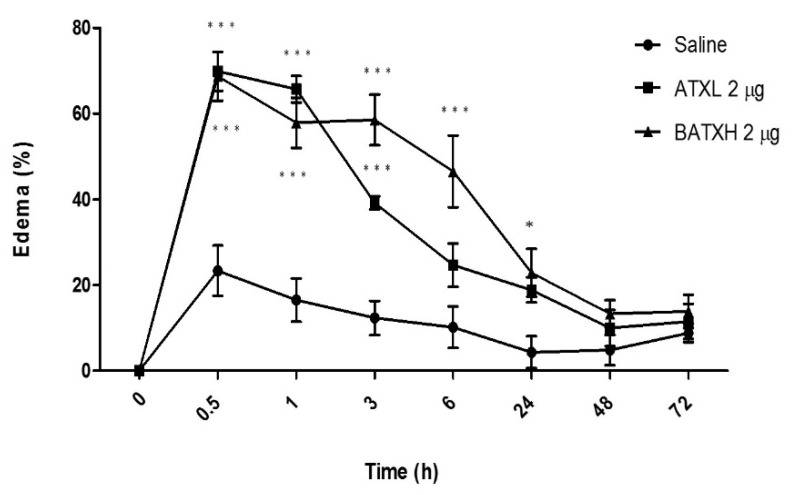
Time course of edema induced by Atroxlysin-Ia (ATXL) and Batroxrhagin (BATXH). Mice (*n* = 6) were injected on the left paw with 2 μg/animal or with saline as negative control. The results are expressed as the means ± S.E. of two independent experiments. * (*p* ≤ 0.05), *** (*p* ≤ 0.001) compared to the negative control group.

According to the kinetics experiments, the edema induced by both toxins peaked between 30 min and 1 h after injection (Figure 2). ATXL-induced edema was significantly higher than control within 3 h after the injection. On the other hand, animals injected into the paw with BATXH showed significantly higher peaks of edema between 30 min and 6 h. In this group, the edema remained until 24 h after injection. Edema reduction occurred progressively until almost reaching the basal level in 48 h, when compared to the control group injected with saline.

**Figure 3:**
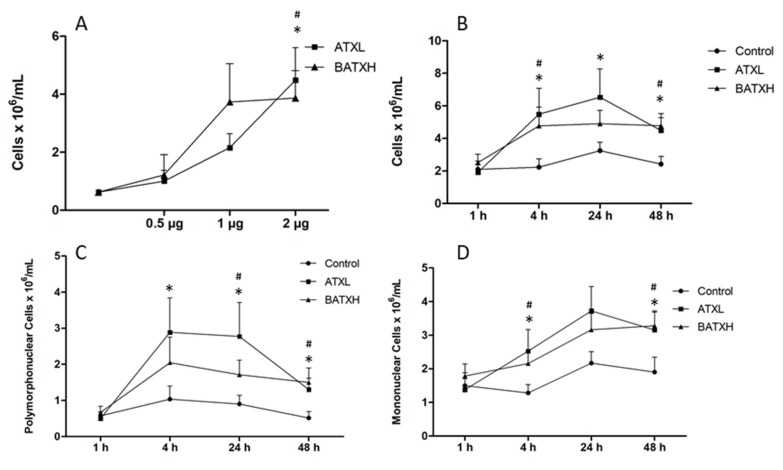
Leukocyte accumulation in the peritoneal cavity induced by Atroxlysin-Ia (ATXL) and Batroxrhagin (BATXH). For dose-response experiments (**A**), mice were injected intraperitoneally with increasing doses of ATXL, BATXH or saline as a negative control, in a final volume of 500 µL/animal. After 4 h, the animals were sacrificed in CO_2_ chamber, for the removal of the peritoneal exudate and the total number of leukocytes counted in Newbauer’s chamber. For time-course experiments, 2 μg of ATXL or BATXH was injected, as described above, and the peritoneal exudate was collected at 1, 4, 24 and 48 h. The total number of leukocytes was counted in Newbauer’s chamber (**B**). Cells were differentiated into polymorphonuclear (**C**) or mononuclear cells (**D**), under optical microscopy (Amplification: 400×). The results are expressed as the mean ± S.E. (*n* = 6–12) of four independent experiments. Significative differences (*p* ≤ 0.05) compared to the negative control for ATXL (*) or BATXH (^#^) groups.

In the following experiment, we compared the leukocyte accumulation induced by intraperitoneal administration of the two SVMPs. In dose-response experiments, doses of 1 and 2 µg of both toxins induced significant leukocyte accumulation, and the differences among 1 and 2 µg doses of both toxin groups were not statistically significant (Figure 3A). The dose of 5 µg/mouse was not tested, as it induced hemorrhage in the previous experiment. The dose of 2 µg/mouse was then selected for the following experiments. In relation to the total number of leukocytes accumulated by the SVMPs in the peritoneum, ATXL induced statistical significant leukocyte influx of 6 × 10^6^ cells/mL at 4, 24 and 48 h after injection, while BATXH showed significant differences from the control after 4 and 48 h, reaching the number of 5 × 10^6^ cells/mL (Figure 3B). In the differential counts, ATXL induced the increase of polymorphonuclear cells in periods of 4, 24 and 48 h after injection, whereas BATXH showed a later response after 24 and 48 h (Figure 3C). Both toxins induced increased influx of mononuclear cells at the periods of 4 and 48 h (Figure 3D).

### 2.2. Cytokines and Chemokine Quantification

We next carried out kinetics assay of inflammatory mediators secreted by Murine Peritoneal Adherent Cells (MPAC) treated with 40 µg/mL *B. atrox* venom, ATXL and BATXH or 1 µg/mL LPS as positive controls, and culture medium, as negative control. Treatments with whole venom or isolated toxins did not induce cytotoxicity in cultures of MPAC, even in incubation periods as long as 24 h (data not shown). MPAC cultures were then submitted to these different stimuli for 2, 4, 6 and 18 h, and the supernatants were analyzed by flow cytometry by using the CBA kit.

**Figure 4:**
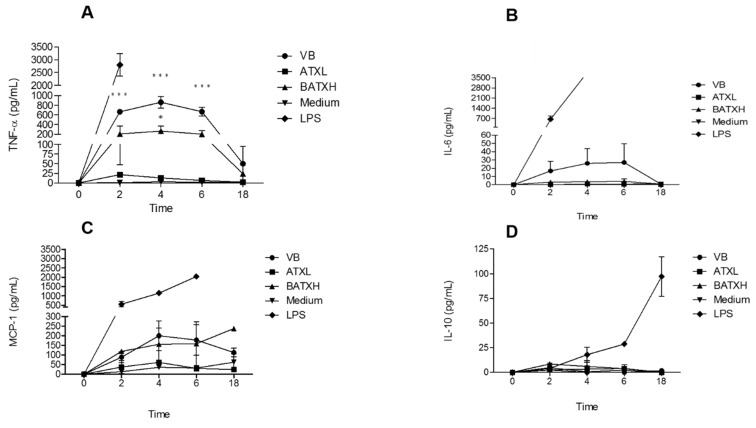
Kinetics of inflammatory mediators secreted by MPACs stimulated with Atroxlysin-Ia (ATXL) or Batroxrhagin (BATXH). MPACs were stimulated with 40 µg/mL of *Bothrops atrox* venom, ATXL, BATXH or LPS (1 µg/mL) as positive control, or culture medium as negative control. Supernatants were collected after 2, 4, 6 and 18 h, for analysis of TNF-α (**A**), IL-6 (**B**), MCP-1 (**C**) and IL-10 (**D**) by the CBA method. The results are expressed as mean ± S.E. of three independent experiments. * (*p* ≤ 0.05), *** (*p* ≤ 0.001) compared to the negative control group.

Figure 4A shows that *B. atrox* venom and BATXH stimulated the secretion of the proinflammatory cytokine TNF-α. Venom-induced TNF-α peaked between 2 and 6 h, with levels up to 800 pg/mL; BATXH induced a significant TNF-α secretion of 200 pg/mL at 4 h. ATXL, on the other hand, showed cytokine levels comparable to the negative control. In our analysis, only the positive control (LPS) induced significant increases of IL-6 and IL-10 cytokines or MCP-1 chemokine in the cell culture supernatants (Figure 4B–D, respectively).

### 2.3. Hydrolysis of Matrigel by SVMPs

**Figure 5:**
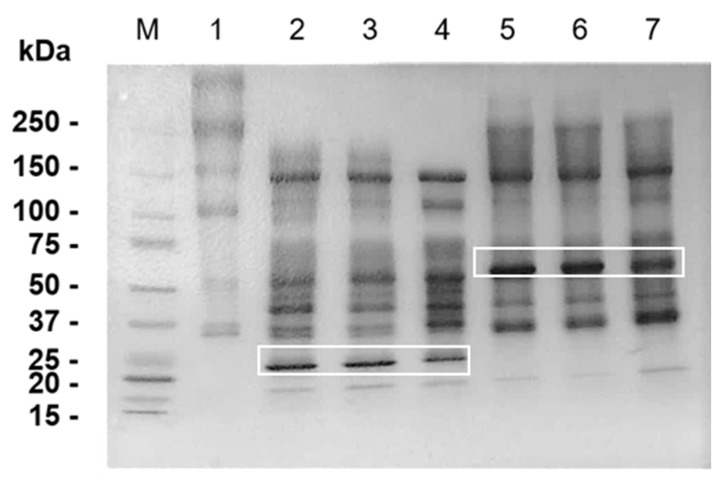
Hydrolysis of Matrigel by Atroxlysin-Ia (ATXL) and Batroxrhagin (BATXH). Matrigel was incubated for different periods of time with the proteinases in the enzyme:substrate ratio of 1:10 (*w*/*w*) at 37 °C. After incubation, the samples were filtered in centrifugal filter devices (cut off at 10 kDa), and the proteins retained in the molecular filters were submitted to SDS-PAGE (5–15% SDS-polyacrylamide gel), under reducing conditions. After electrophoresis, bands were fixed and stained with Coomassie Blue R-250. M—Molecular mass markers; 1—Matrigel (control); 2—ATXL + Matrigel (30 min); 3—ATXL + Matrigel (1 h); 4—ATXL + Matrigel (24 h); 5—BATXH + Matrigel (30 min); 6—BATXH + Matrigel (1 h); 7—BATXH + Matrigel (24 h). The bands of 24 and 52 kDa, indicated by white rectangles, correspond to ATXL and BATXH, respectively.

In order to test our hypothesis that BM peptides generated by SVMP hydrolysis participate in venom-induced inflammation, our first attempt was to check by SDS-PAGE the hydrolysis of Matrigel components after 30 min, 1 h and 24 h of incubation with ATXL and BATXH. In Figure 5, lane 1, we show the Matrigel control with the two major diffuse bands that may include Laminin α-1 (~400 kDa), β and γ (~250 kDa) chains [28], and Collagen IV α-1 (~210 kDa) and α-2 (~190 kDa) chains [7]. Total hydrolysis of the 400 kDa band occurred in the first 30 min of incubation with ATXL (lanes 2–4) and BATXH (lanes 5–7). The 250 kDa band was digested by ATXL in all incubation times, whereas hydrolysis by BATXH provoked only a reduction in the intensity of this band, though it was more evident in the longer incubation time. In the Matrigel control sample, the ~150 and 110 kDa bands correspond to the molecular masses of Nidogen chains [28]. Both SVMPs degraded the 100 kDa band; however, there was no apparent digestion of the 150 kDa band. The highlighted bands of 52 and 25 kDa correspond to ATXL and BATXH, respectively. Interestingly, although the proteinases generated different profiles of Matrigel hydrolysis products, both generated large fragments of 30–220 kDa.

To gain further insight into the hydrolysis of Matrigel components by ATXL and BATXH, we identified the resulting peptide fraction by LC–MS/MS (peptidome). Supplementary Table S1 shows the list of peptides identified with Posterior Error Probability ≤0.01 in the control and proteinase-incubated Matrigel samples. This analysis showed the presence of peptides from various types of proteins in the Matrigel control sample, including intracellular, extracellular and plasma proteins. Moreover, many peptides present in the control Matrigel samples were not detected in those incubated with the proteinases, indicating that they might have been further degraded by the venom proteinases (Supplementary Table S2). The peptidome data were further filtered to only accept peptides that were detected in both LC–MS/MS runs of products derived from Matrigel incubated with each toxin and were absent in the control Matrigel sample runs (Table 1). Accordingly, a rather low number of peptides (17 peptides for ATXL and 10 for BATXH) were detected as hydrolysis products. Laminin α-1 subunit peptides were detected among the products generated by both ATXL and BATXH, while peptides from Laminin β and γ were identified only in the ATXL samples. The low number of peptides identified is in good agreement with the SDS-PAGE profile that showed various protein bands, indicating that the proteinases interacted with Matrigel components, promoting limited proteolysis rather than unspecific degradation to small peptides. Most peptides generated by the proteinases contain a hydrophobic amino acid residue at the N-terminus, including various peptides that contain N-terminal Leu or Ile, which is in agreement with the preference of SVMPs for Leu at the P1′ position of peptides bonds [29] (Table 1).

### 2.4. Characterization of Paw Edema and Leukocyte Accumulation Induced by Matrigel-Derived Peptides

**Figure 6:**
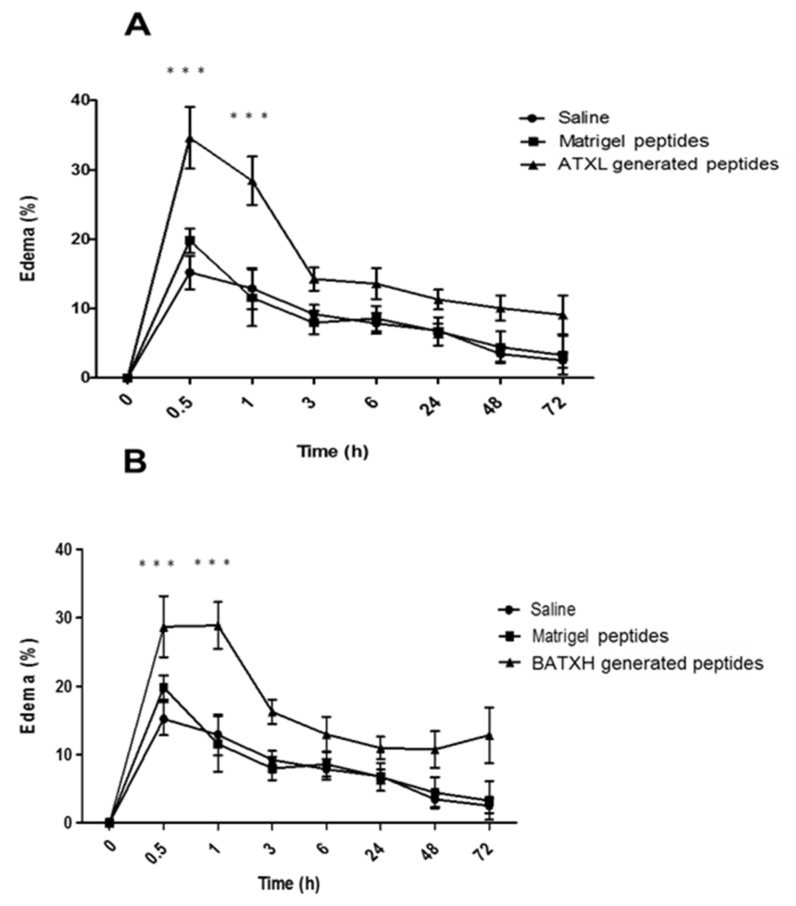
Time course of edema induced by peptides resulting from the hydrolysis of Matrigel by Atroxlysin-Ia (ATXL) (**A**) and Batroxrhagin (BATXH) (**B**). Matrigel was incubated with SVMPs at an enzyme:substrate ratio of 1:10 (*w*/*w*) or with no enzyme, for 1 h, at 37 °C. After the incubation period, the samples were filtered, dried and resuspended in saline solution before injection (30 μl) into the left paw of mice (*n* = 6). The filtrate of a Matrigel sample treated under the same conditions, but without the enzymes (Matrigel peptides), was added to the experiments. Results are expressed as mean ± S.E. of two independent experiments. *** (*p* ≤ 0.001) compared to the Matrigel peptide control group.

We next evaluated the proinflammatory effect of peptides generated by ATXL or BATXH hydrolysis of Matrigel. For this purpose, ATXL or BATXH were incubated with Matrigel for 1 h, and the peptide fraction was isolated by filtering on 10 kDa cut-off membranes, as described in the Methods section. The doses injected in each mouse corresponded to the total amount of peptides released from Matrigel by 10 µg of SVMPs, which is the dose that induced hemorrhage in mice models [20]. Figure 6A,B shows that the products resulting from the hydrolysis of Matrigel by BATXH and ATXL were able to induce edema significantly higher than those of peptides present in Matrigel control samples. The edema was most pronounced in the periods of 30 min to 1 h after injection and reached basal levels around 3 h after injection. Peptides released from Matrigel by ATXL and BATXH caused an increase in the mouse paw of approximately 30–35%, whereas Matrigel peptides induced edema in levels comparable to saline.

**Figure 7:**
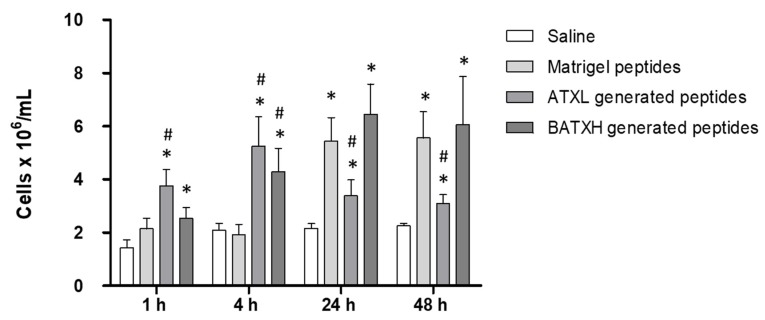
Time course of leukocyte accumulation in the peritoneal cavity induced by peptides released by Atroxlysin-Ia (ATXL) or Batroxrhagin (BATXH) from Matrigel. Mice were injected intraperitoneally with 30 μL of a filtrate resulted from the hydrolysis of Matrigel by ATXL or BATXH. As controls, we used the same volume of filtrates from Matrigel sample treated under the same conditions (Matrigel peptides), but without the enzymes, or with saline only. After 1, 4, 24 and 48 h, the animals were sacrificed in a CO_2_ chamber, for the removal of the peritoneal exudate. The results are expressed as the mean ± S.E. (*n* = 5–6) of two independent experiments. Symbols indicate significative differences (*p* ≤ 0.05) compared to the negative control of saline (*) or Matrigel peptides control group (^#^).

To test the induction of leukocyte accumulation, mice were injected with peptides, as described for the characterization of the paw edema. In this assay, peptides released by ATXL hydrolysis of Matrigel caused a significantly higher leukocyte accumulation of 4–5 × 10^6^ cells/mL, 1 and 4 h after injection, compared to Matrigel control peptides. As for BATXH, a significant increase in the number of leukocytes was observed at 4 h with 4 × 10^6^ cells/mL. In the later periods of 24 and 48 h, Matrigel control peptides induced leukocyte influx of approximately 5 × 10^6^ cell/mL, which is statistically higher than the control levels observed by saline injection. Peptides released by BATXH hydrolysis of Matrigel induced influx of approximately 6 × 10^6^ cell/mL, at 24 and 48 h periods, statistically higher than the saline control, but with no significative difference to the influx induced by the Matrigel control peptides. Peptides resulted from ATXL hydrolysis of Matrigel-induced leukocyte influx levels similar to saline control and significantly lower than Matrigel control peptides, in periods of 24 and 48 h (Figure 7).

**Figure 8:**
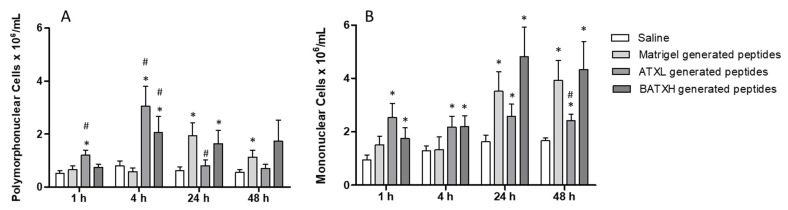
Differential count of leukocytes accumulated in the peritoneal cavity by Atroxlysin-Ia (ATXL) or Batroxrhagin (BATXH). Cells were identified as polymorphonuclear (**A**) or mononuclear (**B**) cells under optical microscopy (Amplification: 400×). The results are expressed as the mean (*n* = 5–6) of two independent experiments. Symbols indicate significative differences (*p* ≤ 0.05) compared to the negative control of saline (*) or Matrigel peptides control group (^#^).

In differential counts, when comparing to Matrigel control peptides, the peptides resulting from the hydrolysis of Matrigel by ATXL induced a significative increase in the accumulation of polymorphonuclear cells after 1 and 4 h and peptides resulted from BATXH hydrolysis of Matrigel only at 4 h (Figure 8A). In the same periods, increases of mononuclear cells by peptides generated by hydrolysis of Matrigel by both toxins were not statistically significant comparing to Matrigel control peptides (Figure 8B). As observed for total leukocyte countings, in periods of 24 and 48 h, the number of polymorphonuclear cells (PMN) and mononuclear cells (MN) accumulated by injection of peptides from the Matrigel control was higher than saline control or peptides generated by Matrigel hydrolysis by ATXL and similar to peptides generated by BATXH hydrolysis of Matrigel.

Thus, we considered as significant only the inflammatory reaction induced 1 and 4 h after injection of peptides released by ATXL and BATXH from Matrigel. Moreover, treatment of MPAC cultures with these peptide fractions did not induce an increase in cytokine or chemokine levels in comparison to the control samples (data not shown).

## 3. Discussion

SVMPs present in the venoms of *Bothrops* snakes are responsible for several inflammatory responses such as edema formation, leukocyte infiltration and the release of mediators such as cytokines and chemokines, which play an important role in the inflammatory process [17]. To compare the proinflammatory action of SVMPs, we tested the local effects of one representative of PI-class (ATXL) and one of the PIII-class (BATXH) SVMPs, both recently isolated by our group from *B. atrox* venom. Both displayed edematogenic activity at low doses ranging from 1 to 5 μg, peaking from 30 min to 1 h, comparable to the other SVMPs isolated from venoms of *Bothrops* snakes [5,30,31]. The injury time of ATXL was similar to that observed with BaP1 [5]. However, in relation to PIII-class SVMPs Jararhagin and BpirMP, BATXH edema was shown to be more persistent [30,31]. It is known that edema formation is influenced by vasoactive amine histamine and eicosanoids such as prostaglandins, which act on vasodilation and increase in the vascular permeability. In normal tissues, the production of prostaglandins is usually low, but in damaged tissues, a large production occurs that precedes the leukocyte recruitment and the process of infiltration of these cells [32,33]. Recently, De Toni et al. [34] observed that the edema in rats generated by Batroxase is mediated by histamine and LTB_4_. In in vitro tests, this metalloproteinase was able to induce degranulation of mast cells, explaining in part the relation of the lesion with the presence of the mediators.

In the leukocyte accumulation induction experiments, we observed an increase in cell infiltrates in the peritoneal cavity induced by the injection of the two selected SVMPs. ATXL induced a slightly larger accumulation compared to BATXH, although not statistically significant. A significant increase of polymorphonuclear cells was observed at 4, 24 and 48 h periods by ATXL, and at 24 and 48 h by BATXH. Curiously, mononuclear cells were also observed a few hours after the induction of the reactions, but the number of mononuclear cells was higher in late periods, such as 24 and 48 h after induction with both toxins. These results are distinct than the tests performed with Batroxase, which demonstrated induction of lower levels of leukocyte influx (2 × 10^6^ cells/mL), with increased influx of polymorphonuclear cells between 2 and 4 h, and increased infiltrate of mononuclear cells in 24 h [17] and data obtained by Fernandes et al. [35] with influx of mononuclear cells only in later periods after injection with BaP1. However, Moreira et al. [15], in agreement with our data, demonstrated the accumulation of mononuclear cells between 1 and 8 h after the induction by *B. atrox* venom, and this accumulation was attributed to the synergistic effect of the different toxins that make up the total venom. The SVMPs used in this study were also able to induce a rapid influx of mononuclear cells that could be explained due to the presence of resident cells, indicating that further analyses are necessary to fully understand the proinflammatory mechanisms of whole venoms and isolated SVMPs.

Interestingly, in spite of the structural differences between ATXL and BATXH, both toxins induced inflammatory signs at the same magnitude. The catalytic domain present on both ATXL and BATXH is involved in the degradation of ECM molecules [7], activation of pro-MMPs [10], processing of cytokines as pro-TNF-α [11] or proteins from the complement system [36]. These effects are responsible for induction or amplification of inflammatory reaction, including the cytokine release, and may be essential mechanisms involved in the proinflammatory reaction of SVMPs. Studies using MMP inhibitors have already confirmed the importance of the catalytic activity for the proinflammatory activity of SVMPs in experimental mice models. Leukocyte infiltrates induced by Jararhagin or BaP1 were reduced by treatments with chelating agents, such as o-phenanthroline or EDTA, respectively [8,35]. Escalante et al. [5] tested the action of Batimastat, a broad-spectrum synthetic inhibitor of MMPs, in local damage induced by BaP1 and observed a reduction in bleeding, dermonecrosis and edema in mouse models. More recently, Preciado et al. [37] demonstrated the efficacy of the synthetic CP 471474 inhibitor in edema induced by Batx-I, isolated from *B. atrox* venom. These observations indicate the relevance of catalytic activity for the proinflammatory activity of SVMPs. However, the importance of disintegrin-like and cysteine-rich domains of PIII-class SVMPs, present only in BATXH, has also been reported. Catalytically inactivated PIII-class SVMPs are still able to induce leukocyte recruitment [8] and mRNA synthesis of proinflammatory cytokines [12], thus implying a direct interaction with different receptors attributed to motifs present on the disintegrin-like [38], cysteine-rich domains [39] or both [13].

It has been demonstrated that the inflammatory response induced by the venom of *B. atrox* activates TLR2, which plays a role in the migration of polymorphonuclear cells [40], such as neutrophils, which are the first cells of the immune system to migrate to the site of inflammation, where they are effective in the elimination of pathogens and the production of cytokines [41]. Thus, in order to better understand the mechanisms involved in ATXL and BATXH induction of inflammation, we performed kinetic assays for the quantification of inflammatory mediators commonly found in these events. Our tests showed that MPAC cells secreted the cytokine Tumor Necrosis Factor alpha (TNF-α) when stimulated with *B. atrox* venom or BATXH, while ATXL did not induce synthesis of this mediator. In similar tests with MPAC cells, Clissa et al. [12] reported that Jararhagin, a PIII-class SVMP, induced TNF-α release, while Rucavado et al. [42] did not identify TNF-α in the culture of cells stimulated with a PI-class SVMP, the BaP1 from *B. asper* venom, indicating that the disintegrin-like and/or cysteine-rich domains present only in the PIII-class SVMPs may be acting directly on cellular receptors, thus explaining the lack of stimuli after incubation with PI-class SVMPs.

Interleukin-6 (IL-6), MCP-1 and IL-10 were not identified in the tests performed with ATXL or BATXH, and levels similar to the negative control were found in the samples incubated with the whole venom. IL-6 plays an important role during inflammation process through activation of polymorphonuclear cells, regulation of adhesion molecules, differentiation of T cells and synthesis of other cytokines [43,44], and it was already shown that different PI- and PIII-class SVMPs are able to stimulate IL-6 secretion [13,31,45]. Thus, the apparent contradiction between our observations with previous reports may be attributed to a proteolytic degradation of secreted IL-6 by the metalloproteinase activity of the enzymes, in the same fashion as previously reported in a study that used similar protocol [12].

It is known that one of the best described mechanisms of action of SVMPs is the hydrolysis of BM components, as demonstrated in in vitro and in vivo assays [46]. Baldo et al. [47] demonstrated that Jararhagin and the Jararhagin-C (devoid of catalytic domain) have an affinity for collagen IV of basal membrane and bind to tissues accumulating around the capillary vessels. Later, using in vitro assays with 2D and 3D culture models of HUVECs, it was observed that Jararhagin co-localized on the surface or was internalized by endothelial cells, and also visualized on the surface of the tubules formed in the 3D cultures [48]. The binding of Jararhagin to different substrates of the extracellular matrix as collagen IV and Matrigel and to α_2_β_1_ integrin was also reported [29,47]. The basement membrane is a complex structure that is formed by several molecules, such as collagen IV, laminin and nidogen, among other components, and acts as a network, guaranteeing stability to blood vessels and capillaries. Furthermore, the exacerbated hydrolysis of ECM components generate a large amount of fragments that could have a secondary action on the local lesion that occurs in snakebites [27]. Heparan sulphate proteoglycans (HSPG) are also structural components of ECM that modulate adhesion and transmigration of leukocytes to the injury site [49,50]. It is very likely that the hydrolysis of these components releases small fragments acting on some mechanisms of inflammation. ECM fragments can act as damage-associated molecular patterns (DAMPs) by interacting with pattern recognition receptors (PRRs), such as Toll-like receptors (TLRs), triggering a series of proinflammatory events from the innate immune response, which may subsequently influence the adaptive immune response [51,52]. DAMPs can influence the modulation of proinflammatory mediators, cell activation, differentiation and proliferation, and stimulate regeneration processes [53,54].

Recently, it was demonstrated that the muscular exudate of CD-1 mice injected with 50 μg of *B. asper* venom was able to increase the vascular permeability of other animals injected with this material. A subproteomic analysis of this material revealed the presence of more cytokines and chemokines in the collected material 24 h after venom injection, in detriment to the exudate of 1 h, indicating that, even after venom diffusion, these mediators continue to be produced and may be related to the inflammatory processes caused during the envenoming [27]. Thus, we suggest that ECM fragments released by the proteolytic action of SVMPs from *B. atrox* venom, particularly ATXL and BATXH, could act as DAMPs and trigger endogenous stimuli similar to those occurring in cell death processes. To test this hypothesis, we evaluated whether the products of Matrigel hydrolysis by ATXL or BATXH had proinflammatory effects. Matrigel is mostly composed of collagen IV, laminin, nidogen and heparan sulphate proteoglycan. After incubating it with ATXL and BATXH, we observed complete hydrolysis by both SVMPs of the band of approximately 400 kDa, which may correspond to laminin α-chain [28]. However, only ATXL was able to fully hydrolyze the lower molecular mass band (~250 kDa), which corresponds to the molecular masses of laminin β- and γ-chains and collagen IV [7]. Nidogen [28] was partially degraded by the two SVMPs. In the LC–MS/MS analysis, peptides corresponding to laminin α-, β- and γ-chains were identified in the Matrigel samples incubated with ATXL, while BATXH generated peptides from the laminin α-chain only. Previous reports have already shown differential hydrolysis of BM components by PI- and PIII-class SVMPs. Escalante et al. [28] demonstrated the extensive hydrolysis of laminin α and γ chains by BaPI (PI-class) and in the case of Jararhagin (PIII-class) by most of hydrolysis was of the nidogen and only the α-chain of laminin. In in vivo experiments, Freitas-De-Sousa et al. [20] observed that ATXL and BATXH degraded collagen IV and laminin, with total collagen digestion by ATXL, which would explain the proteinase ability to induce hemorrhage, since these BM components play important role in the stability of capillaries.

The products resulting from the hydrolysis of the Matrigel by ATXL and BATXH were then tested for proinflammatory activity. Both samples induced a fast edema formation and peritoneal cell infiltrates in levels statistically significant when compared to Matrigel control peptides. The leukocytes found in the peritoneum region in these periods/conditions were mostly polymorphonuclear cells. Between 24 and 48 h, the number of cells accumulated by the control Matrigel peptides not exposed to SVMPs hydrolysis was higher than those of the saline control and similar to those of the BATXH-generated peptides, respectively. Commercial Matrigel is widely used in human stem cell cultures by mimicking the extracellular matrix (ECM) [55], and it is known to contain components other than structural ECM proteins, such as growth factors and MMPs 2 and 9 [56], or even intracellular or membrane proteins [55]. Talbot et al. [57] demonstrated by semi-quantitative analyses, with capture antibody arrays, the presence of various secreted or soluble proteins and peptides classified as growth factors, chemokines and other proteins with biological activity in four different lots from commercial Matrigel, including the Vascular Endothelial Growth Factor (VEGF), chemokine MCP-1 and peptide C5a. However, these results did not answer some questions about the biological activity of proteins or peptides present in Matrigel, which could impact experiments in vitro involving cell culture. The presence of these bioactive components in Matrigel may explain the fact that our control sample composed of Matrigel peptide fraction (without enzyme digestion) induced a leukocyte accumulation in the peritoneum of injected animals in periods of 24 and 48 h. However, the opposite was observed in the edema induction tests and in the early phase of the leukocyte accumulation test. In these situations, Matrigel peptides did not evoke any inflammatory activity, and this result leads us to suggest that peptides specifically liberated by incubation with ATXL or BATXL are causing the observed early events of the inflammatory reaction.

Other important aspect to be discussed is that only peptides with molecular masses smaller than 10 kDa were used in our model, to induce inflammatory reaction. According to our results of electrophoresis, hydrolysis products were spotted in bands between 30 and 220 kDa, indicating that the effects of ATXL and BATXH on Matrigel correlate more to limited proteolysis rather than unspecific degradation to small peptides. Herrera et al. [58] identified dozens of fragments derived from extracellular matrix proteins, including heparan sulphate proteoglycan, collagen IV and nidogen, by means of proteomic analysis of the exudate extracted from the gastrocnemius muscle of mice injected with the *B. asper* venom. Considering that larger BM protein fragments also act as DAMPs and these fragments were not included in our sample, it is reasonable to expect that more evident proinflammatory effects will be observed when we test the intermediate molecular mass fragments. Based on these indications, we suggest that the peptides released after the degradation of the BM components by ATXL and BATXH are stimulating cell-receptors as part of the inflammatory process and acting indirectly on the endogenous metalloproteinases, amplifying the inflammatory response observed in the experiments.

In conclusion, ATXL and BATXH SVMPs isolated from *B. atrox* venom are capable of inducing edema formation and leukocyte recruitment in experimental models; however, they do so with slightly different profiles. ATXL induced a higher leukocyte accumulation throughout the evaluation periods, while BATXH induced MPACs for cytokine release as observed with whole venom, particularly of TNF-α. Both metalloproteinases hydrolyzed major components of Matrigel, generating products of different sizes. Once more, ATXL hydrolysis was more extensive, and all Laminin chains were completely degraded and generated peptides identified by LC–MS/MS. The products resulting from SVMP hydrolysis of Matrigel were capable of inducing edematogenic activity and leukocyte accumulation in levels significantly higher than Matrigel control peptides. These data indicate that proinflammatory action of SVMPs may occur by different a mechanism, including the participation of hydrolysis products generated from their catalytic action on BM components.

## 4. Materials and Methods

### 4.1. Toxins

*Bothrops atrox* venom was provided by the Herpetology Laboratory, Instituto Butantan, collected from the snakes maintained under captivity at the Institute for venom production. Atroxlysin-Ia (ATXL) and Batroxrhagin (BATXH) were isolated from *B. atrox* venom, according to the methodologies described previously [20,21].

### 4.2. Hydrolysis of Matrigel Components and Isolation of the Resulting Peptides

Matrigel obtained from the Engelbreth–Holm–Swarm murine sarcoma (Sigma-Aldrich, St Louis, MO, USA) was incubated with ATXL or BATXH at an enzyme:substrate ratio of 1:10 (*w*/*w*) in 10 mM Tris-HCl buffer, pH 7.4, for 30 min, 1 or 24 h, at 37 °C. After the incubation period, the reaction was stopped by adding one additional volume of the same ice-cold buffer, and the samples were processed in two different ways: (1) For mass spectrometry, proteins remaining in the hydrolysis solution (1 µg enzyme:10 µg Matrigel in 40 µL) were precipitated by the addition of 8 volumes of ice cold acetone and 1 volume of ice cold methanol and stored for 12 h, at −20 °C. Then the peptides were recovered from the supernatants, after centrifugation for 10 min, at 14,000× *g*, at 4 °C. (2) For electrophoresis and biological assays, after digestion, the hydrolysis mixtures (80 µg enzyme:800 µg Matrigel in 1 mL) were placed in centrifugal filter devices (cut off at 10 kDa) (Amicon Ultra-2, Merck, Darmstadt,, Germany) and centrifuged at 1500× *g*, for 15 min, at 15 °C, until the volume of 500 µL. Aliquots of sample retained in the filter were analyzed by electrophoresis in a 5–15% gradient polyacrylamide gel. The filtered solutions (500 µL) were dried in a SpeedVac concentrator (Thermo Fisher Scientific, Waltham, MA, USA) and suspended in 120 μL of ice-cold saline. Of these, 30 μL was injected into each mouse, for in vivo assays. In this way, the amount of peptides injected into each mouse corresponded to the amount of peptides that were expected to be generated by 10 μg of ATXL or BATXH, which is the dose at which they induce hemorrhage in mice [20].

### 4.3. Gel Electrophoresis

For the analysis of the material retained on the centrifugal filter devices, SDS-PAGE was performed by using 5–15% acrylamide gradient gel according to the technique described by Laemmli [59], but with some modifications. The fractions were diluted 1:3 in sample buffer (350 mM Tris-HCl, 10% SDS, 30% glycerol and 1.2 mg/mL bromophenol blue), under reducing conditions (DTT-Dithiothreitol 9.3% in sample buffer), boiled for 5 min and applied to a stacking gel with 4% polyacrylamide. Electrophoresis occurred with constant amperage of 35 mA and voltage of 180 V, using run buffer (25 mM Tris; 190 mM Glycine, 0.1% SDS, pH 8.3). The gels were stained with Coomassie R-250 blue 0.25% in 25% methanol and 5% acetic acid) and decolorized with bleach solution (40% methanol + 7% acetic acid). Precision Plus Protein Kaleidoscope molecular mass standard (M.W. 10–250 kDa, BIO-RAD, Hercules, CA, USA) was used.

### 4.4. Analysis of Generated Peptides by LC–MS/MS

The supernatant containing the peptide fraction obtained from the hydrolysis of Matrigel by ATXL and BATXH, as described in Section 4.2, was dried in a SpeedVac vacuum concentrator and dissolved in 0.1% TFA, for desalting, using Sep-pak C-18 cartridges previously conditioned with methanol and 0.1% TFA (Waters, Milford, MA, USA). Samples were dried and reconstituted in 0.1% formic acid (solution A), and injected in EASY Nano LCII system (Thermo Scientific, Waltham, MA, USA), into a 5 cm of 10 μm Jupiter C-18 trap column (100 μm I.D. × 360 μm O.D.) coupled to an LTQ-Orbitrap Velos mass spectrometer (Thermo Scientific). Chromatographic separation was performed on a 15 cm long column (75 μm I.D. x 360 μm O.D.). Elution occurred with a linear gradient of 5–40% acetonitrile in 0.1% formic acid (solution B) in 60 min, at 200 nL/min. Spray voltage was set at 2.4 kV, and the mass spectrometer was operated in data-dependent mode, in which one full MS scan was acquired in the *m*/*z* range of 400–2000, followed by MS/MS acquisition, using higher energy collision dissociation (HCD) of the ten most intense ions from the MS scan. MS and MS/MS spectra were acquired in the Orbitrap analyzer, at 60,000 and 7500 resolution (at 400 *m*/*z*), respectively. The maximum injection time and AGC target were set to 25 ms and 1E6 for full MS, and 250 and 100 ms and 5E4 for MS/MS. The minimum signal threshold to trigger fragmentation event, isolation window and normalized collision energy (NCE) were set to, respectively, 5000 cps, 2 *m*/*z* and 40. Dynamic peak exclusion was applied to avoid the same *m*/*z* of being selected for the next 90 s. Two independent LC–MS/MS runs were performed for each sample.

Mass spectrometric raw data were analyzed by using MaxQuant software (version 1.5.3.12; Cox and Mann, 2008), using the UniProt protein sequences of *Mus musculus* (16,619 sequences, reviewed; date of fasta file: 8 February 2016). The search parameters were as follows: no enzyme specificity; deamidation of glutamine and asparagine and oxidation of methionine were considered as variable modification; peptide and MS/MS mass tolerances were set to 10 ppm and ±0.025 Da, respectively. Data from both LC–MS/MS runs were filtered by Posterior Error Probability value ≤0.01 for each peptide (Supplementary Table S1). Data were further filtered to only accept peptides that were detected in both technical replicates and were absent in the control samples (Supplementary Table S2).

### 4.5. Evaluation of the Inflammatory Reaction Induced by ATXL, BATXH and Matrigel Hydrolysis Products

#### 4.5.1. Animals

Male mice of the BALB/c strain (18–20 g) were used in the experiments and kept under controlled temperature and light periods with water and feed ad libitum. All experiments were approved by the Butantan Institute Ethics Committee on Animal Use (Protocol Number: 6708040817), on 22 August 2014.

#### 4.5.2. Paw Edema

The protocols described by Kimura et al. [32] and Távora et al. [60] were used for evaluation of edema induction, but with some modifications. In the tests, mice (*n* = 6/group) had the left hind paw measured with pachymeter (Starret), prior to injection of the samples (zero time). Subsequently, the concentrations of 0.5, 1, 2 and 5 μg of the toxins diluted in 30 μL of saline or 30 μL of the peptide solution, obtained as described in Section 4.2, were injected into the plantar pad of the same foot. Animals injected with saline were used as control. The injected paws were measured again at different time intervals (30 min, 1, 3, 6, 24, 48 and 72 h), and the results were expressed as the percent of increase between the measurements of the paw at the experimental and zero time. Animals injected with saline or Matrigel treated at the same conditions, but without incubation with SVMPs, were used as control groups.

#### 4.5.3. Leukocyte Recruitment

ATXL and BATXH or filtrates from Matrigel hydrolysis by SVMPs, obtained as described in Section 4.2 (30 µL), were diluted in 500 μL of sterile saline and injected intraperitoneally into groups of 6–12 animals/group. A group injected with saline alone or filtrates obtained from Matrigel, without SVMPs, were used as control. The animals were euthanized in the CO_2_ chamber after 1, 4, 24 and 48 h, and the peritoneum was washed with 2 mL of ice-cold saline solution. The peritoneal exudate was centrifuged at 500× *g* at 4 °C for 6 min. After centrifugation, the pellet was resuspended in saline and diluted 1:10 (*v*/*v*) in the Turk’s solution, with some modifications (600 µL of acetic acid + 2 mg of methyl violet in the final volume of 20 mL distilled water), and cells were counted in Newbauer’s chamber. In the differential count, the cells were classified as polymorphonuclear and mononuclear, according to the morphological differences. The total and differential counts were performed under an optical microscope, with increases of 400×.

### 4.6. Production of Inflammatory Mediators

Cytokines and chemokines released by treatment with whole venom or isolated SVMPs were assayed in supernatants of stimulated MPAC cultures extracted from BALB/c mice. Animals were euthanized in a CO_2_ chamber, and the peritoneum was washed and massaged with 1.5 mL of RPMI 1640 culture medium (Gibco–Life Technologies, Waltham, MA, USA) supplemented with 10% fetal bovine serum. (Gibco–Life Technologies). The obtained exudate was centrifuged at 1500× *g* for 10 min at 4 °C. The supernatant was discarded, and the cells were suspended in 2 mL of supplemented culture medium. The cells were counted in a Newbauer’s chamber and subsequently placed in 96-well plates (6 × 10^4^ cells / well), which were then incubated at 37 °C, in the presence of 5% CO_2_, for 24 h. Non-adherent cells were removed by PBS washing, and cells that adhered to the plate were used in the cell viability assay and inflammatory mediator quantitation assay. Cultures were tested for the toxicity by the MTT (3- (4,5-dimethylthiazol-2-yl) 2,5-diphenyl tetrazolium bromide) cleavage assay of mitochondrial-cell enzymes, resulting in the formation of formazan blue. MPAC cells were stimulated with the nontoxic concentrations of 40 µg/mL of ATXL, BATXH or *B. atrox* venom, LPS (1 µg/mL) (Sigma-Aldrich, St. Louis, MO, USA), or incubated with culture medium only, as negative control. Supernatants were collected at 2, 4, 6 and 18 h after the start of incubation.

TNF-α, IL-10 and IL-6 cytokines levels, and the MCP-1 chemokine in the cell culture supernatant, were quantified on the equipment FACSCantoII (BD Biosciences, Franklin Lakes, NJ,, USA), using the Cytometric Bead Array (CBA) Mouse Inflammation Kit (BD Biosciences, USA), according to the manufacturer’s recommendations.

### 4.7. Data Analysis

Student’s *t*-test was performed to evaluate differences between two groups. Differences between three or more groups were assessed by ANOVA, followed by Bonferroni’s test. The analyses were performed by using the software GraphPad Prism 5.0 (San Diego, CA, USA).

## Figures and Tables

**Table 1:** Peptides generated by the incubation of Matrigel with Atroxlysin-Ia (ATXL) and Batroxrhagin (BATXH).

Proteins *	Uniprot Entry	Identified Peptides *	
		ATXL	BATXH
Laminin subunit alpha-1	P19137	HADIIIKGNG	ALLHAPTGS
		IRSQQDVLGGHRQ	LWDLGSGSTR
		LVEHVPGRPVR	LINGRPSADDPSP
		LINGRPSADDPSP	
Laminin subunit beta-1	P02469	AIKQADEDIQGTQN	
Laminin subunit gamma-1	P02468	IRNTIEETGI	
Tubulin beta-4B chain	P68372	HSLGGGTGSGMGT	
Vimentin	P20152	ANYQDTIGR	
Actin, cytoplasmic 2	P63260	TVLSGGTTMYPGIAD	
		QVITIGNER	
Fibrinogen beta chain	Q8K0E8	LRPAPPPISGGGY	
60 kDa heat shock protein, mitochondrial	P63038	VGGTSDVEVNEK	
60S ribosomal protein L30	P62889	IIDPGDSDIIR	
Glyceraldehyde-3-phosphate dehydrogenase	P16858	HSSTFDAGAGIA	IFQERDPTNIK
			ITIFQERDPTNIK
Heterogeneous nuclear ribonucleoprotein F	Q9Z2X1	SVQRPGPYDRPGTA	
Prolyl 3-hydroxylase 1	Q3V1T4	FSSGTENPHGVKA	
Transcription intermediary factor 1-beta	Q62318	LTEGPGAEGPR	
40S ribosomal protein S3	P62908		IGPKKPLPDHVS
40S ribosomal protein S4, X isoform	P62702		TIRYPDPLI
78 kDa glucose-regulated protein	P20029		VAFTPEGER
Hemoglobin subunit beta-1	P02088		LVVYPWTQR
Protein disulfide-isomerase	P09103		ITSNSGVFSK

* Protein group full description, and all data on biological replicates, including criteria to accept peptide identification, are shown in Materials and Methods and Supplementary Tables S1 and S2.

## Supplementary Materials

The following are available online at https://www.mdpi.com/2072-6651/12/2/96/s1. Table S1. Identification of peptides generated by the incubation of Matrigel with ATXL and BATXH by LC–MS/MS; Table S2. Proteins present in Matrigel and identified as cleaved by ATXL and BATXH by LC–MS/MS.
